# Physical Inactivity Is Associated With Increased Levels of Anxiety, Depression, and Stress in Brazilians During the COVID-19 Pandemic: A Cross-Sectional Study

**DOI:** 10.3389/fpsyt.2020.565291

**Published:** 2020-11-17

**Authors:** Lucas Raphael Bento Silva, Camila Simões Seguro, Camila Grasiele Araújo de Oliveira, Paulo Otávio Silva Santos, Jordana Campos Martins de Oliveira, Luiz Fernando Martins de Souza Filho, Célio Antônio de Paula Júnior, Paulo Gentil, Ana Cristina Silva Rebelo

**Affiliations:** ^1^Department of Physical Education, University Center Araguaia, Goiânia, Brazil; ^2^Postgraduate Program in Health Sciences, Faculty of Medicine, Federal University of Goiás, Goiânia, Brazil; ^3^Faculty of Physical Education and Dance, Federal University of Goiás, Goiânia, Brazil; ^4^Department of Morphology, Institute of Biological Sciences, Federal University of Goiás, Goiânia, Brazil

**Keywords:** anxiety, depression, stress, COVID-19, physical exercise, mental health, pandemic, physical inactivity

## Abstract

**Objective:** To evaluate the levels of anxiety, depression, and stress associated with the practice of physical exercise (PE) during pandemic by COVID-19.

**Methods:** This study has a cross-sectional characteristic and was carried out between May 12 and 14, 2020. An online questionnaire was applied with questions to assess sociodemographic characteristics and physical exercise during the CoVID-19 pandemic, in addition to depression, anxiety, and stress analysis. The study was approved by the local ethics committee (CAAE: 31521720.8.0000.5082).

**Results:** One thousand one hundred and fifty four answered the questionnaire (69.84% female). During the isolation period, the number of participants who declared not to exercise was 54.16%. Women generaly presented higher levels of anxiety, depression, and stress when compared to men (*p* < 0.0001 for all domains). The risk of having increased anxiety were 118% higher (OR = 2.183; 95% CI = 1.717–2.775), the risk of depression was 152% higher (OR = 2.525; 95% CI = 1.991–3.205), and the risk of stress symptoms increased 75.1% (OR = 1.751; 95% CI = 1.386–2.213) in the participants who did not perform PE when compared to those who maintain regular PE.

**Conclusion:** People who was not involved with PE during the COVID-19 pandemic had higher anxiety, depression, and stress scores. Based on this, it seems important to advise people to continue PE, following all the recommendations of preventive measures of the pertinent health organizations.

## Introduction

In December 2019, the World Health Organization (WHO) officially reported cases of pneumonia with no apparent cause, in the province of Hubei in China, the new disease caused by a type of coronavirus was designated with COVID-19 (1). COVID-19 quickly crossed borders, infecting people around the world, and was considered a pandemic in early March (2). Currently there are officially 5,438,837 confirmed cases and 340,585 deaths by the new virus. At the moment, Brazil stills in an upward curve of contamination, in May 24, 2020 there were 347,398 cases of infected with 22,013 numbers of deaths (https://covid.saude.gov.br).

Widespread outbreaks of infectious diseases, such as COVID-19, are associated with emotional and psychological distress and symptoms of mental disorders, with a higher prevalence of anxiety, depression, and stress (3–7). The number of people affected by these symptoms tends to be higher than the number of people affected by the infection (8, 9). Limited knowledge about COVID-19, lack of update on COVID-19, duration of home confinement, poor physical health and physical symptoms, lack of access to healthcare, lack of activity, uncertainty regarding the economic scenario and health systems can increase anxiety and depression (3, 6, 10, 11). In addition, social isolation—working and studying at home, having dependent children, decreased physical contact with other people—can be a significant psychological stressor (12, 13) and cause negative lifestyle changes such as poor diet and physical inactivity (14).

Physical exercise (PE) has been associated with improved psychological outcomes (15); and its neurobiological effects seem to influence several neural mechanisms related to depressive and anxiety disorders (16, 17). The absence or reduction of PE has been associated with an increased risk of mental disorders such as anxiety and depression (18–20). Although there is not yet an ideal dose of exercise for this type of diseases, there is evidence demonstrating that any exercise is better than none on mental disorders (15, 20, 21). Studies show that the regular practice of PE can be compared to pharmacological measures for the treatment of mental disorders such as depression (22, 23).

However, in the midst of the global public health crisis scenario, little is known about the relationship between PE and the attenuation of symptoms of mental disorderscaused by the current pandemic. Morover, it would be important to know if physcial activity habits would be related to markers of stress, anxiety, and depression. In view of the above, the objective of this study was to evaluate the levels of anxiety, depression, and stress associated with the practice of PE during COVID-19 outbreak.

## Methods

### Study Design and Participants

This study has a cross-sectional characteristic and was conducted between May 12 and 14, 2020, the invitation to participate in the study was made through social media to reach the largest number of respondents. All participants were informed of the evaluation model to be applied and consented to participate. The study was approved by the local ethics committee (CAAE: 31521720.8.0000.5082).

### Procedures

A questionnaire was applied via online platform to all those who showed interest to participate in the survey, using the Google® forms system. The questionnaire was divided into two parts, the first involved demographic characteristics (age, gender, marital status, and educational level), weight and height for BMI calculation, history of diseases, PE practice before and during COVID-19 outbreak, fear of contamination and prevention measures adopted.

In the second part of the questionnaire, mental health status was assessed using the Depression Anxiety Stress Scale (DASS-21) (24), based on previous studies (25, 26). The scale consists of 21 self-reports items that assess the leve of anxiety (items 2, 4, 7, 9, 15, 19 e 20), depression (items: 3, 5, 10, 13, 16, 17 e 21) and stress (items 1, 6, 8, 11, 12, 14 e 18), the answers are according to the Likert scale, four points and pass through points 0 (Did not apply to me at all), 1 (Applied to me to some degree, or some of the time), 2 (Applied to me to a considerable degree or a good part of time), and 3 (Applied to me very much or most of the time).

The DASS-21 scale scores are classified by summing the relevant items, and the cutoff points for the anxiety subscale are: normal (<7), mild (8–9), moderate (10–14), severe (15–19), and extremely severe (>20). For depression subscale: normal (<9), mild (10–13), moderate (14–20), severe (21–27), and extremely severe (>28). The cutoff points for the stress subscale are: normal (<14), mild (15–18), moderate (19–25), severe (26–33), and extremely severe (>34).

### Statiscal Analysis

The continuous variables of the study are presented in mean ± standard deviation and categorical variables were presented in frequency (percentage). The Shapiro-Wilk was used to assess the normality of the continuous variables. The independent sample *t*-test was used to compare anxiety, depression, and stress scores between men and women. The Chi-square test was used to verify the differences between categorical variables. The binary logistic regression was used to assess the association between regular physical exercise and levels of anxiety, depression, and stress. The significance level adopted was *p* < 0.05 and the statistical software used was the Statistical Package for the Social Sciences (SPSS, IBM Corp., Armonk, NY, USA) version 21.0.

## Results

### Participants and Demographics Characteristics

A total of 1,154 responded the questionnaire, among them 69.84% were women. Mean age of the participants was 31.15 ± 9.68 and mean BMI was 25.32 ± 5.05. The majority of the participants were single (women: 55.08% and men: 71.83%, with a statistical difference *p* = 0.042). Only 193 interviewees reported pre-existing diseases, including: cancer (0.49%), diabetes mellitus (1.73%), respiratory diseases (9.61%), and arterial hypertension (5.02).

When asked about PE practice before and during the COVID-19 outbreak, 278 women and 72 men reported not practicing any type of PE before social isolation (30.32% of the participants). During the isolation period the number of participants who stated no PE practicewere 434 women and 191 men representing 54.16% of the total. The other data are presented in Table 1.

**Table 1:** Characteristics of the participants.

	**Total (*n* = 1154)**	**Female (*n* = 806)**	**Male (*n* = 348)**	
	**N (%)**	**N (%)**	**N (%)**	***p*-value**
Age, year				0.116
18–29	604 (52.33)	389 (48.26)	215 (61.78)	
30–45	450 (39.00)	334 (41.44)	116 (33.33)	
>45	100 (8.64)	83 (10.30)	17 (4.89)	
BMI, kg/m^2^				0.397
<24.9	631 (54.67)	460 (57.07)	171 (49.14)	
25.0–29.9	367 (31.80)	235 (29.15)	132 (37.93)	
>30.0	156 (13.53)	111 (13.78)	45 (12.93)	
**Marital status**				
Single	694 (60.13)	444 (55.08)	250 (71.83)	0.042^*^
Married/common-law marriage	402 (34.83)	314 (38.96)	88 (25.28)	
Divorced/widowed	58 (5.04)	48 (5.96)	10 (2.89)	
Educational Level				0.261
Elementary education	73 (6.32)	48 (5.95)	25 (7.18)	
College degree	417 (36.13)	270 (33.50)	147 (42.24)	
Graduate degree	464 (40.20)	358 (44.43)	106 (30.45)	
Not answer	200 (17.35)	130 (16.12)	70 (20.11)	
Reported dieases				
Arterial hypertension	58 (5.02)	40 (4.96)	18 (5.17)	
Cancer	4 (0.49)	4 (0.49)	–	
Diabetes	20 (1.73)	15 (1.86)	5 (1.43)	
None	976 (84.57)	674 (83.62)	302 (86.78)	
Respiratory diseases	111 (9.61)	86 (10.66)	25 (7.18)	
Physical exercise before pandemic				0.057
Yes	804 (69.67)	528 (65.62)	276 (79.31)	
No	350 (30.33)	278 (34.48)	72 (20.79)	
Physical exercise during pandemic				0.127
Yes	569 (49.30)	372 (46.24)	197 (56.79)	
No	585 (50.70)	434 (53.86)	151 (43.31)	

X^2^—test;

* *Statistically significant p < 0.05*.

### Risk of Contamination and Preventive Measures

A considerable percentage of the participants (96.78% of the women and 85.62% of the men) reported having some fear of contamination by COVID-19. Table 2 presents the measures taken by the participants, all reported taking at least two of the measures mentioned.

**Table 2:** Percepction of contamination risk and preventive measures taken by respondents.

	**Female (*n* = 806)**	**Male (*n* = 348)**
	**N (%)**	**N (%)**
**Are you afraid of being infected with the new coronavirus (Covid-19)?**		
Very	470 (58.31)	158 (45.40)
Little	294 (38.47)	140 (40.22)
None	42 (5.22)	50 (14.38)
**What is your chance of being infected by the new coronavirus (Covid-19)?**		
Big	64 (7.94)	35 (10.05)
Medium	275 (34.11)	131 (37.64)
Small	288 (35.73)	111 (31.89)
None	28 (3.47)	24 (6.89)
Don't know to answer	151 (18.75)	47 (13.53)
**Preventive measures**		
Cleaning hands with alcohol gel	691 (85.73)	323 (92.81)
Washin hands with soap	736 (91.31)	296 (85.05)
Using disinfectants at home	546 (67.74)	162 (46.55)
Avoiding leaving home	709 (87.96)	279 (80.17)
Avoiding physical contact	652 (80.89)	278 (79.88)
Avoiding crowded places	756 (93.73)	313 (89.94)
Avoiding touching eyes and nose	581 (72.08)	241 (69.25)

### Anxiety, Depression, and Stress Levels

Women presented higher levels of anxiety, depressoin, and stress when compared to men (*p* < 0.0001 for all domains). It was observed that 374 women (46.41%) and 100 men (28.74%) had mild to extremely severe anxiety levels. In the depression domain, 487 women (60.43%), and 142 men (40.81%) showed mild to extremely severe levels. Regarding stress, 417 women (51.74%), and 110 men (31.61%) presented levels from mild to extremely severe. The frequency per domain is shown in Table 3.

**Table 3:** Number of participants showing psychological symptoms during quarantine by COVID-19, stratified by sex, using DASS-21.

	**Sex**		***p*-value**
	**Female (*n* = 806)**	**Male (*n* = 348)**	
	**N (%)**	**N (%)**	
Anxiety			<0.0001^*^
Normal	432 (53.59)	248 (71.26)	
Mild	61 (7.56)	16 (4.59)	
Moderate	146 (18.11)	49 (14.08)	
Severe	61 (7.56)	13 (3.73)	
Extremaly severe	106 (13.18)	22 (6.34)	
Depression			<0.0001^*^
Normal	319 (39.57)	206 (59.19)	
Mild	124 (15.38)	39 (11.20)	
Moderate	195 (24.19)	54 (15.51)	
Severe	68 (8.43)	27 (7.76)	
Extremaly severe	100 (12.43)	22 (6.34)	
Stress			<0.0001^*^
Normal	389 (48.26)	238 (68.39)	
Mild	112 (13.89)	42 (12.06)	
Moderate	126 (15.63)	34 (9.77)	
Severe	123 (15.26)	26 (7.47)	
Extremaly severe	56 (6.94)	8 (2.31)	

* *Statistically significant p < 0.05*.

### Associations and Influence of Physical Exercise During Quarantine on Anxiety, Depression, and Stress

Table 4 shows the comparison of the general scores of anxiety, depression, and stress among the interviewees according to PE practice during the period of social isolation. Individuals who performed PE had lower levels of anxiety, stress, and depression (*p* < 0.0001 in all domains for women *p* = 0.0103, <0.0001 and 0.0001, respectively, in men) when compared to individuals who were not performing PE during the COVID-19 outbreak.

**Table 4:** Comparison of anxiety, depression, and stress levels in respondents who do or do not physical exercises during quarantine by COVID-19.

	**Female**			**Male**		
	**PA (*n* = 372)**	**PI (*n* = 434)**	***p*-value**	**PA (*n* = 197)**	**PI (*n* = 151)**	***p*-value**
	**N (%)**	**N (%)**		**N (%)**	**N (%)**	
Anxiety			<0.0001			0.0103
Normal	241 (64.80)	191 (44.00)		148 (75.13)	100 (66.22)	
Mild	29 (7.79)	32 (7.37)		10 (5.08)	6 (3.97)	
Moderate	51 (13.70)	95 (61.88)		24 (12.18)	25 (16.55)	
Severe	20 (5.38)	41 (9.44)		3 (1.52)	10 (6.63)	
Extremaly severe	31 (8.33)	75 (17.31)		12 (6.09)	10 (6.63)	
Depression			<0.0001			<0.0001
Normal	188 (50.55)	131 (30.18)		136 (69.04)	70 (46.35)	
Mild	61 (16.41)	63 (14.51)		20 (10.15)	19 (12.58)	
Moderate	70 (18.81)	127 (29.26)		25 (12.69)	29 (19.20)	
Severe	27 (7.25)	39 (9.00)		8 (4.06)	19 (12.60)	
Extremaly severe	26 (6.98)	74 (17.05)		8 (4.06)	14 (9.27)	
Stress			<0.0001			0.0001
Normal	203 (54.59)	186 (42.85)		146 (74.12)	92 (60.92)	
Mild	60 (16.12)	52 (11.98)		30 (15.23)	12 (7.94)	
Moderate	58 (15.59)	68 (15.69)		11 (5.58)	23 (15.26	
Severe	40 (10.75)	83 (19.12)		6 (3.04)	20 (13.24)	
Extremaly severe	11 (2.95)	45 (10.36)		4 (2.03)	4 (2.64)	

*PA, physically active during pandemic for Covid-19; PI, physically inactive during pandemic for Covid-19*.

Table 5 shows the association between psychological symptoms and PE practice, adjusted for age, in the total population, between women and men. The risk of having increased anxiety were 118% higher (OR = 2.183; 95% CI = 1.717–2.775), the risk of depression was 152% higher (OR = 2.525; 95% CI = 1.991–3.205) and the risk of stress symptoms increased 75.1% (OR = 1.751; 95% CI = 1.386–2.213) in the participants who did not perform PE when compared to those who maintain regular PE. Among women, the chance of showing symptoms of anxiety and depression was more than two times greater (OR = 2.341; 95% CI = 1.760–3.112 and OR = 2.363; 95% CI = 1.771–3.154, respectively) for those did no perform PE. Absence of PE was related to 60% higher risk of having elevated stress levels (OR = 1.602; 95% CI = 1.212–2.117). In men, there was a greater risk of having higher levels of anxiety, depression, and stress (OR = 2.929; 95% CI = 1.911–4.449; 4.045; 95% CI = 2.657–6.155 and 3.247; 95% CI = 2.147–4.993, all presented *p* < 0.0001) in those who did not perform PE.

**Table 5:** Association between physical exercise and mental health status measured by DASS-21, adjusted for age and stratified for sex.

		**Anxiety**			**Depression**			**Stress**		
		**OR**	**95% CI**	***p*-value**	**OR**	**95% CI**	***p*-value**	**OR**	**95% CI**	***p*-value**
All	Physically active during quarantine	1.00			1.00			1.00		
	Physically inactive during quarantine	2.183	1.717; 2.775	<0.0001	2.526	1.991; 3.205	<0.0001	1.751	1.751; 2.213	<0.0001
Female	Physically active during quarantine	1.00			1.00			1.00		
	Physically inactive during quarantine	2.341	1.760; 3.112	<0.0001	2.363	1.771; 3.154	<0.0001	1.602	1.212; 2.117	0.001
Male	Physically active during quarantine	1.00			1.00			1.00		
	Physically inactive during quarantine	2.929	1.911; 4.490	<0.0001	4.045	2.657; 6.155	<0.0001	3.247	2.147; 4.993	<0.0001

*OR, odds ratio; 95% CI, 95% confidence interval*.

## Discussion

A recent scientometric analysis found that the most common research topics include emergency care and surgical, viral pathogenesis, and global responses in the COVID-19 pandemic but there is a lack of research on PE (27). Our results showed an increase of 67.14% in the number of people who did not perform PE during the COVID-19 pandemic. Women had higher scores of anxiety, depression, and stress when compared to men. Our analysis showed that thosewho did not practice PE showed higher values in all subscales evaluated (anxiety, depression, and stress). After binary logistic regression, the lack of PE practice was associated with higher values of anxiety, depression, and stress in both men and women.

Our results showed that individuals who performed PE during coronavirus outbreak presented lower levels of anxiety, stress and depression. When considering both sexes, those who did not perform PE had a 118% higher risk of presenting symptoms of anxiety, 152% more chance of having values of depression above normal and 75.1% higher risk of having symptoms of stress. Women who did not perform PE were 134% more likely to have high anxiety scores, 136% more likely to have depressive symptoms and 66% more likely to show high levels of stress. As for men, those who were not involved with PE were 192, 304, and 224% more likely to have high levels of anxiety, depression, and stress, respectively, as shown in Table 5.

Our results are in agreement with previous studies. Fluetsch et al. (28) evaluated the “The 2015 Behavioral Risk Factor Surveillance System” and found an inverse relationship between PE and mental health for those who reported being insufficiently active. Teychenne et al. (29) reported that sedentary behavior was associated with increased risk of anxiety. A meta-analysis documented that PE interventions significantly improved depressive symptoms among healthy adults (30). Moreover, it has been shown that PE have the potential to decrease the self-reported days of mental health problems due to anxiety, depression, and stress (28). Schuch et al. (31), when performing a meta-analysis with prospective studies (at least 1 year of follow-up), showed that higher levels of self-reported PE are associated with a lower risk of anxiety symptoms and anxiety disorders when compared to lower levels of PE.

The relationship between PE and mental health problems seems to be bidirectional. Exercise reduce the risk of anxiety and/or depression symptoms, but these symptoms may also lead the individual perform PE. Da Silva et al. (32), reported that regular PE was associated with a lower probability of depressive symptoms; but in the inverse analysis, participants with symptoms of anxiety and depression were more likely to not reach the recommended levels of PE. However, there are possible mechanisms that might explain the influence of PE on mood. These mechanisms though might involve the release of endorphins (33), thermogensis (34), the activation of the mTOR axis in specific brain regions (35) and neurotransmitters such as dopamine and serotonin (36, 37).

In the current scenario, measures aiming to control covid-19 dissemination require social isolation and restrictions that might agravate mood symptoms. However, the present results suggest that PE may decrease the risk of depression, anxiety, and stress. Therefore, it might be advisable to recommend the performance of PE, obviously respecting safety recommendations as part of the behavior therapy (38) and health education (39) during the COVID-19 pandemic.

The WHO recommends 150 min of PE for asymptomatic people, which can be distributed throughout the week, and for those people with comorbidities who do not present symptoms the recommendation is to continue with active habits (40) From a practical standpoint, PE can be performed with numerous possibilities. It is possible to adapt materials, use body weight exercises, elastic bands, exercise with no external loads, calisthenics and others (41–49).

The study has some important limitations. We opted to perform a simple analysis of PE habits (yes or no question). Whist this facilited response, it does not precisely define different levels, so we cannot stablish a dose response relation. Another important limitation is that the mode of delivery (electronic) leaded mostly younger adults that have access to internet to respond; therefore, it might not reflect the situation of groups with different socioechonomical and age status. It might not reflect the situation of groups with different socioechonomical and age status. This study did not explore the use of face mask as preventive measures which was found to be associated with lower prevalence of depression (7) but cannot be used during PE.

## Conclusion

Based on the present results, it is possible to note a reduction in regular PE during social isolation. Moreover, people who was not involved with PE during the COVID-19 pandemic had higher anxiety, depression, and stress scores. Based on this, it seems important to advise people to continue PE, following all the recommendations of preventive measures of the pertinent health organizations.

## Data Availability Statement

The raw data supporting the conclusions of this article will be made available by the authors, without undue reservation.

## Ethics Statement

This study involved human participants and was reviewed and approved by the Research Ethics Committee linked to the Center for Excellence in Teaching, Research and Projects Leide das Neves Ferreira of the National Health Council. The patients/participants provided their written informed consent to participate in this study.

## Author Contributions

LRBS: concept and study design, collected and analyzed data, wrote, reviewed, and edited the manuscript. CS and CO: collected data, wrote, reviewed, and edited the manuscript. PS, JO, LFMS, and CP: collected data and edited the manuscript. PG and AR: contributed with data analysis, reviewed, and edited the manuscript. All authors contributed to the article and approved the submitted version.

## Conflict of Interest

The authors declare that the research was conducted in the absence of any commercial or financial relationships that could be construed as a potential conflict of interest.
